# IgG4 related pericardium and lung disease in pediatric patient complicated with fatal massive hemoptysis: a case report and review of literature

**DOI:** 10.1186/s12969-023-00799-7

**Published:** 2023-02-13

**Authors:** Moustafa Soliman, Hamdy Ahmed, Rasmia Elgohary, Hala Ibrahem El Gendy

**Affiliations:** 1 Rheumatology and Clinical Immunology Unit, Internal Medicine Department, Kasr Alainy Faculty of Medicine, Cairo University, Cairo, Egypt; 2 Division of Rheumatology, Department of Medicine, University of South Alabama, Mobile, AL USA

**Keywords:** Pediatric Immunoglobulin G-4 related disease, Pulmonary IgG4-RD, Pericardial IgG4-RD, IgG4-RD

## Abstract

**Background:**

IgG4-related disease (IgG4-RD) is a progressive and sometimes fatal disease that rarely affects pediatric age group. It may affect the orbits, lacrimal and salivary glands, pancreas, kidneys, peritoneum and other organs. Lung and pleura are not commonly reported in IgG4-RD. We here present a rare case of pediatric IgG4-RD with rare involvement of pericardium, pleura and lungs.

**Case presentation:**

A 13-year-old girl presented with intrathoracic IgG4-RD with pleuropericardial involvement. She showed initial improvement on prednisolone. Azathioprine and then mycophenolate failed to control relapses during steroid tapering. Her last relapse was treated by rituximab however, the patient developed acute fatal massive hemoptysis.

**Conclusions:**

Pediatric IgG4-RD is a rare entity with pericardio-pulmonary affection as the rare of the rare. Usual treatment of prednisolone and steroid sparing agents should be used, with rituximab used as a rescue therapy, but fatal complications may occur.

## Background

IgG4-related disease (IgG4-RD) is a progressive, destructive and sometimes fatal disease. It can present with enlargement of the involved organ that may affect the orbits, lacrimal and salivary glands, pancreas, kidneys, lungs, pleura, peritoneum and other organs, and appropriate clinico-pathological findings sometimes supported with high IgG4 level are needed for diagnosis [1], according to the updated diagnostic criteria published in 2020 [2].

Treatment usually includes oral prednisolone with gradual tapering over a long time while adding a steroid sparing agent like: azathioprine, mycophenolate mofetil and B cell depleting therapy as rituximab [3].

Lung and pleura are not commonly reported in IgG4-RD with the percentage estimated as 15% of cases in some reports [4].

Epidemiology of IgG4-RD in pediatric population is not well studied and the data is usually retrieved from case reports and case series. A recent Turkish single center study identified 8 pediatric cases with IgG4-RD with equal number of both males and females and median age of 13.4 years and pulmonary manifestations in only one case [5].

In this case report, we describe a case of 13-year-old girl presenting with intrathoracic IgG4-RD which is a rare manifestation of a rare disease in a population rarely affected by this disease.

## Case presentation

A 13-year-old previously healthy female student presented with gradual exertional dyspnea that improved on leaning forward. The condition progressed over 2 months and was associated with two attacks of coughing blood-tinged sputum, night fever, decreased appetite and weight loss. She was discovered to have a massive pericardial effusion with no clinical or radiographic evidence of tamponade. Therapeutic pericardiocentesis revealed an exudative fibrinous effusion with marked leukocytosis, predominant polymorphonuclear lymphocytes and abundant lympho-plasmacytic cells. Its culture and sensitivity were negative. A Chest Computed Tomography (CT) showed marked pericardial effusion with mild pericardial thickening, left pleural basal thickening in addition to bilateral patchy areas of pulmonary interstitial thickening (consolidation) and ground glass veiling.

An extensive work up showed normal complete blood count (CBC), elevated erythrocyte sedimentation rate (ESR) and C-reactive protein (CRP), normal liver function tests apart from hypoalbuminemia, normal kidney function tests, negative tuberculin test, negative blood culture and sensitivity, negative anti-nuclear antibodies, rheumatoid factor and virology, normal complement 3 and 4 and normal thyroid profile, the workup was negative for respiratory viruses and bacteria. She received empirical antibiotics and a short course of steroids and was discharged home.

Despite initial improvement, shortness of breath, hemoptysis, fever and weight loss recurred, and she was readmitted. On examination she appeared toxic, orthopneic, tachypneic, febrile (temp; 38.5 °C-39°C) and tachycardic with no evidence of pulsus paradoxus. Her neck veins were congested and non-pulsating. Her cardiac examination showed increased dullness of the bare area of the heart, distant heart sounds, no murmur or pericardial rub. Bilateral scattered crackles and wheezes were apparent on chest auscultation. The patient had no lower-limb oedema, hepatomegaly or lymphadenopathy.

**Fig. 1:**
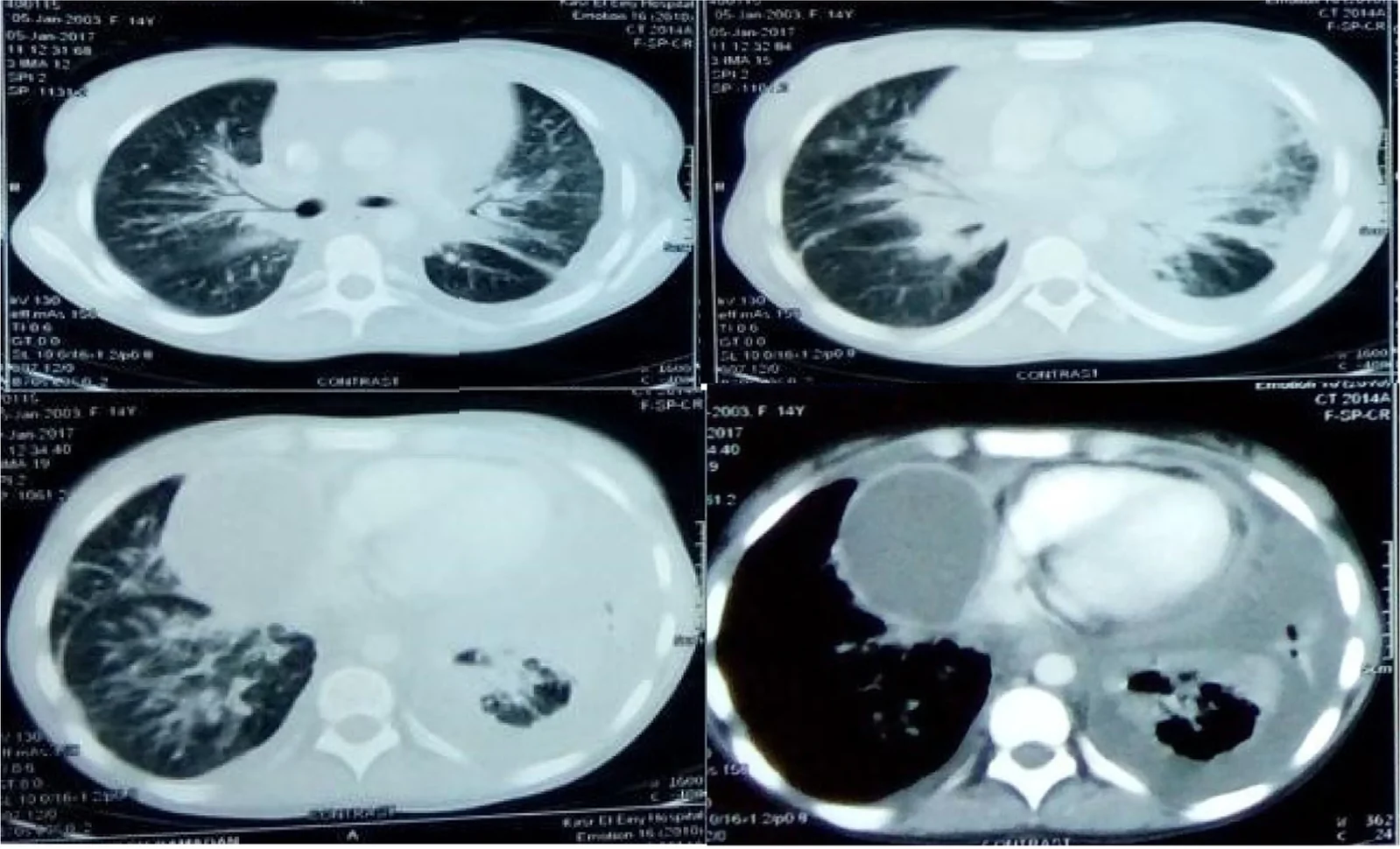
CT chest: lung window showed consolidation with air bronchogram in lung midzone and lower lobes. Mediastinal window (lower right) showed massive pericardial effusion, encysted effusion

The investigations including diagnostic pericardiocentesis, and CT-chest (Fig. 1) were similar to the initial admission except for normocytic anemia. In addition three early-morning sputum samples smears with Ziehl–Neelsen (ZN) stain, Interferon-Gamma Releasing Assay (IGRA) test and sputum culture were negative.

The patient received vancomycin and ceftriaxone, with marked improvement. Due to extensive adhesions pericardiotomy was recommended. The encysted pericardial fluid was aspirated. Pericardiotomy was done with multiple biopsies taken from the pericardium. The patient tolerated the procedure without postoperative complications.

Pericardial fluid analysis showed predominant lymphocytes. The Mycobacteria Growth Indicator Tube (MGIT) culture for tuberculosis (TB) was negative after 2-months incubation.

**Fig. 2:**
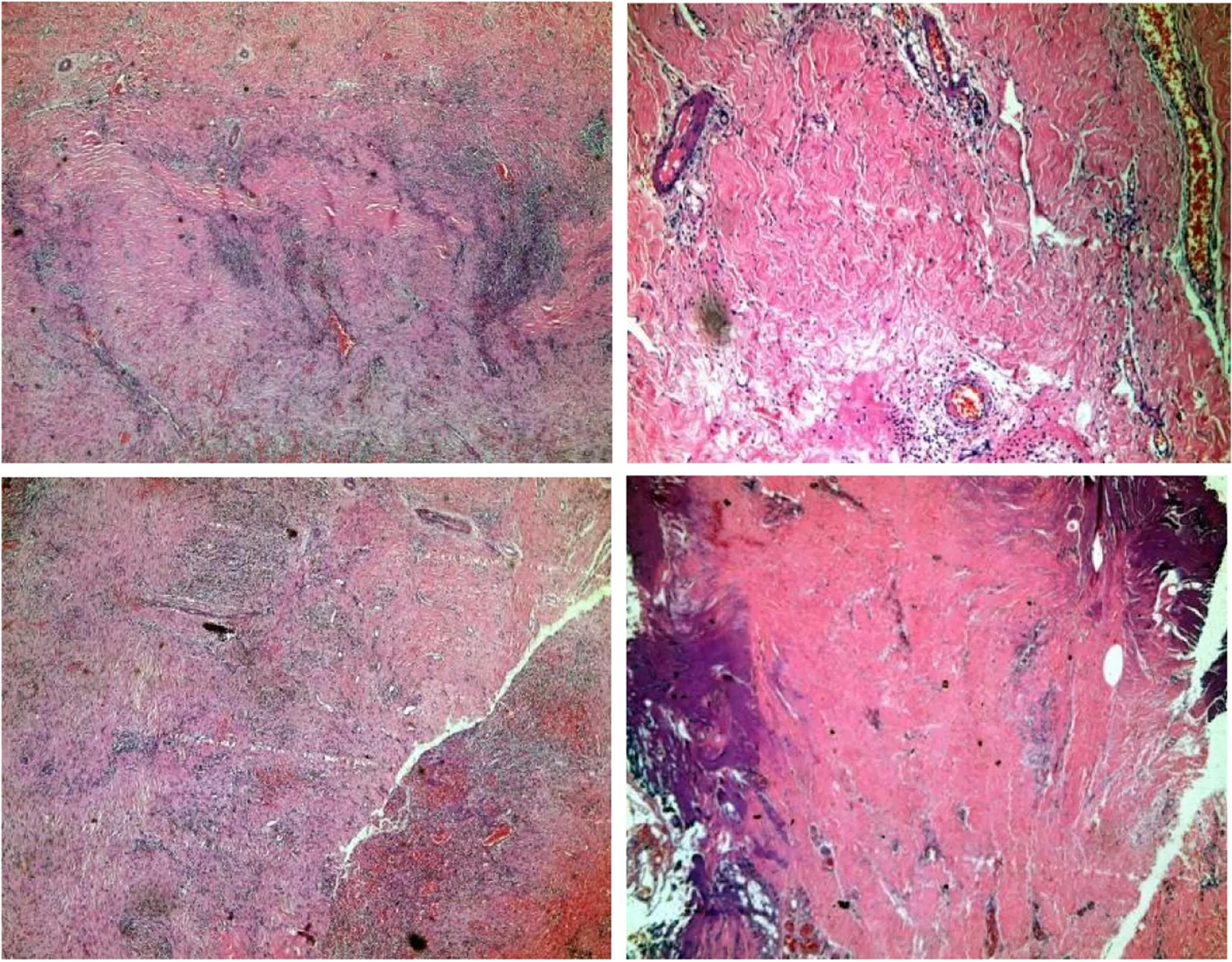
Pericardial pathology showed storiform fibrosis with excess lympho-plasmacytic infiltrate

**Fig. 3:**
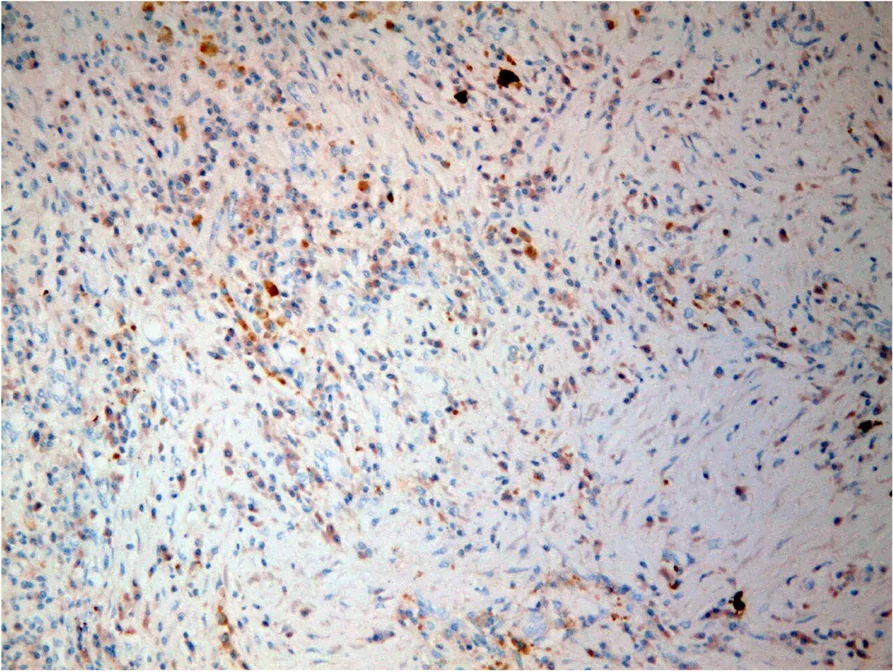
The immune-histochemistry showed increased number of IgG4-positive plasma cells findings was compatible with IgG4 related disease

Pericardial Pathology (Figs. 2 and 3) revealed marked storiform fibrosis with excess lymphoplasmacytic infiltrate. The immune-histochemistry showed an increased number of IgG4-positive plasma cells, findings that were compatible with IgG4 related disease. Serum IgG: 2600 mg/dl (700–1600), Serum IgG4: 168 mg/dl (40–120), (value for IgG4-RD > 135 mg/dl), Serum IgG4/total IgG: 0.064 (cut off value for IgG4-RD > 0.08).

Thus, the patient was diagnosed with IgG4 related disease affecting her pericardium and lungs. She was started on 0.6 mg/kg prednisone together with 2.5 mg/kg azathioprine. After 2-weeks the patient returned to her usual physical activity. After three months, there was no recurrence of pericardial or pleural effusion, with normalization of hemoglobin, ESR and CRP. However, the lung lesions persisted and she was switched to mycophenolate mofetil (MMF) and rituximab was discussed.

2 years later, the patient started to complain of progressive dyspnea, with high ESR, CRP, so the decision was to start rituximab. The patient received 4 intravenous doses of rituximab 500 mg, each dose was one week apart from its following dose. After those four doses, the patient showed marked initial improvement of her general condition, dyspnea and cough.

6 months later, the patient started to redevelop progressive dyspnea, and productive cough with whitish sputum, culture and sensitivity were negative, the patient was not feverish, the chest auscultation showed left basal inspiratory crepitations. The hemoglobin level was 9.4 mg/dL, normal white blood cell count and differential, normal basic metabolic profile, ESR = 16, CRP was negative.

**Fig. 4:**
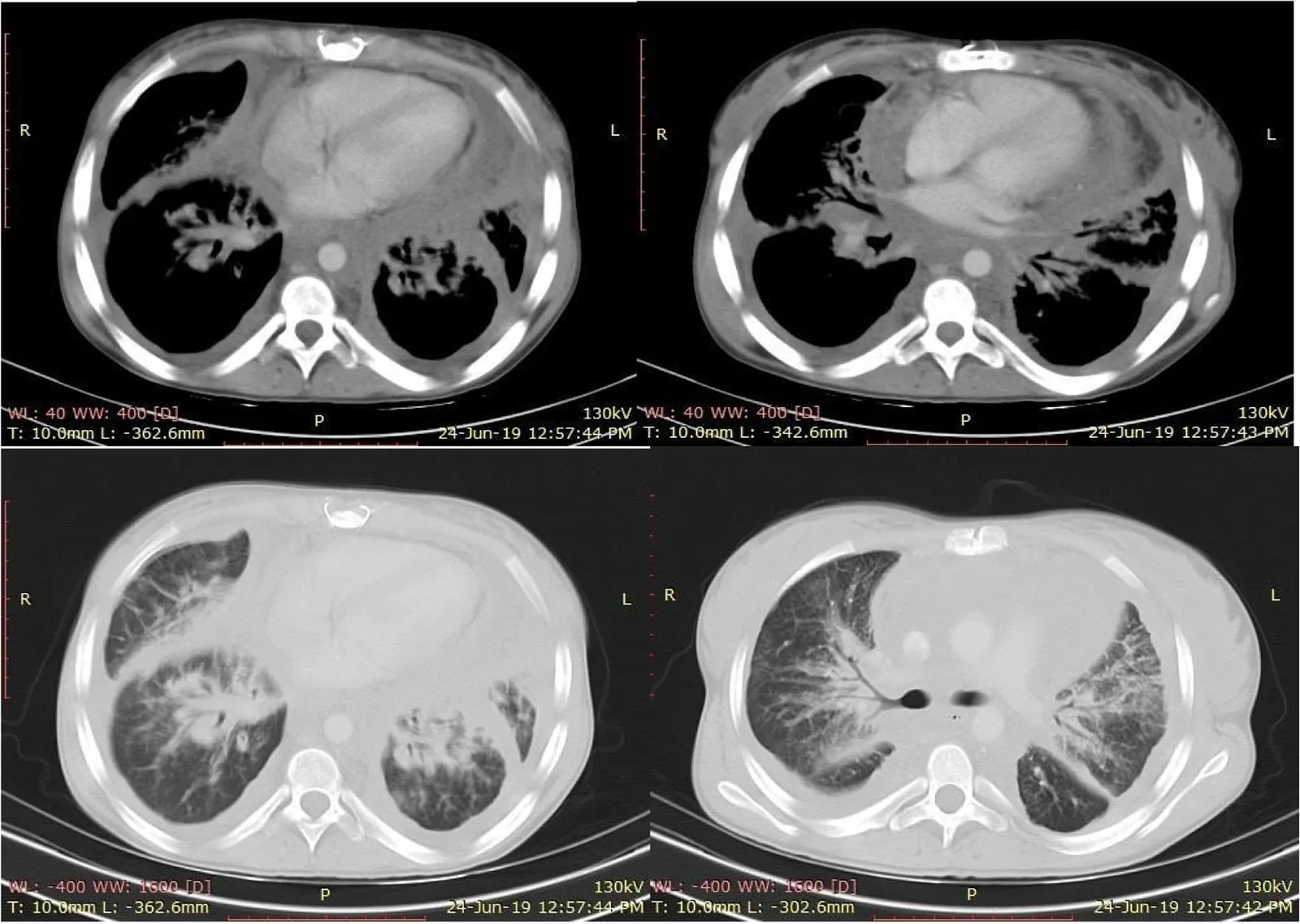
CT chest showing diffuse mediastinal infiltrative soft tissue lesion, pericardial thickening with effusion, bilateral diffuse thickening of central and peripheral pulmonary interstitium

CT chest with intravenous contrast (Fig. 4) was done and showed: diffuse mediastinal infiltrative soft tissue mass lesion is seen surrounding the mediastinal structures with no evidence of obstruction, associated with bilateral circumferential pleural and fissural thickening, more evident on the left side, with bilateral pleural effusion, there was also pericardial thickening with effusion. There was bilateral diffuse thickening of central and peripheral pulmonary interstitium in the form of hilar and peribronchovascular soft tissue thickening and thickening of interlobular septae.

Echocardiography showed normal ejection fraction, EPAP = 30 mmHg, no pericardial involvement.

Left thoracotomy was done, superior mediastinal and pleural biopsies were taken, the pathology showed: inflammatory fibrosing reaction with no active IgG-4 related disease in the tissue examined.

The patient received one dose of intravenous rituximab 500 mg and oral prednisolone was increased to 1 mg/kg/day and the patient was discharged for follow-up.

**Fig. 5:**
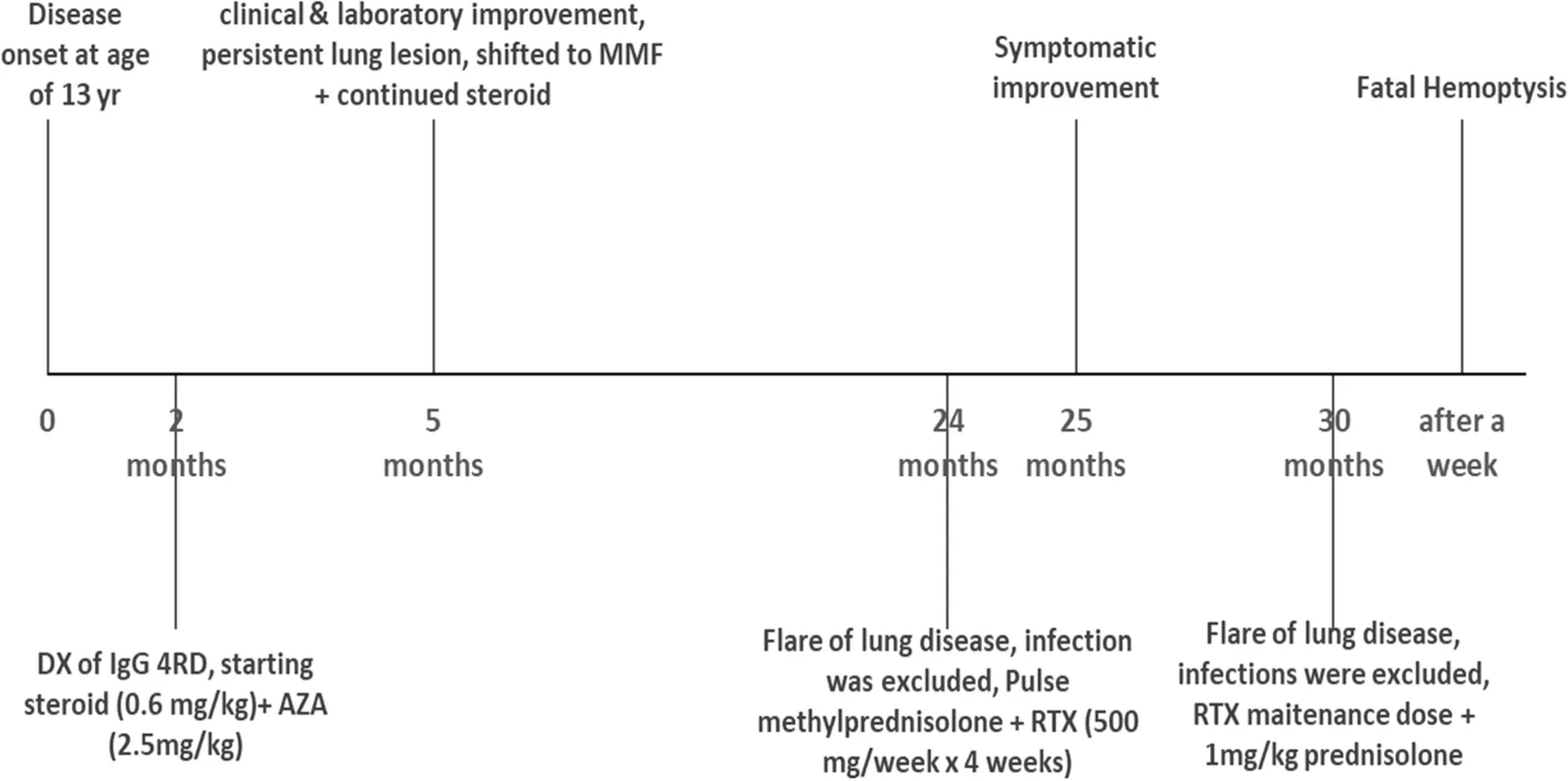
Timeline of the events. (Yr: years, Dx: diagnosis, IgG4-RD: Immunoglobulin G 4 related disease, AZA: azathioprine, MMF: mycophenolate mofetil, RTX: rituximab)

One week later the patient developed a sudden onset of massive hemoptysis. She was brought to emergency room with altered mental status, bradycardia, hypotension and hypoxia. She was intubated, and mechanically ventilated but despite resuscitation the patient passed away. The timeline of the events is shown in Fig. 5 (Fig. 5: timeline of the events).

## Discussion and conclusions

This is a case report of a rare presentation of intrathoracic IgG4-RD in a young 13-year-old female. She fulfilled definitive diagnosis according to the revised comprehensive diagnostic (RCD) criteria for IgG4-RD having all three criteria: 1) organ involvement; 2) serum IgG4 concentration > 135 mg/dl; 3) positive for pathological sub-items. Constitutional symptoms and inflammatory markers were evident from the start paralleling the activity of the disease and improved later on with treatment.

We have searched Pubmed using: (IgG4-RD AND pediatric) starting from all the time till the date of July17th, 2022 and this search resulted in identifying 28 cases of IgG4-RD in pediatric age group, along with 23 cases extracted from another review [6] and extra 8 cases from single center experience [5], a total of 59 cases as shown in (Table 1: literature review of pediatric IgG4-RD).

**Table 1:** literature review of pediatric IgG4-RD

Authors	Type of study	No	Age and gender	Main presentation	Other manifestations	Fever/ Constitutional symptoms	Acute phase reactants	Lines of treatment	Serum IgG4 level	Histopathological Confirmation	Methods of follow up	Outcome
(Tille et al., 2020) [9]	Case report	1	16-year-old girl	Orbital inflammation/ Colitis	-	Weight loss but No fever	ESR = 25 mm/hour, normal CRP	Prednisolone and MTX	3.6 g/L (0.05–1.96)	Present	Clinical/APRs	improvement
(Dylewska et al., 2020) [10]	Case report	1	13-year old boy	Tumour of the orbit and pterygopalatine fossa	Lymphadenopathy	Yes	Raised	Prednisone 0.6 mg/kg	350 mg/dL (4–230 mg/dL)	Present	Clinical/APRs/IgG 4 level in serum/Radiological	Complete regression
(Smerla et al., 2018) [11]	Case report	1	4-year-old boy	Orbital inflammation	-	No	Normal	Prednisolone	222 mg/dl (1–189)	Present	Radiological	Complete resolution
(Aydemir et al., 2019) [12]	Retrospective study	6	10 years ± 3—three boys/three girls	Auotoimmune hepatitis	-	-	-	Prednisolone, azathioprine	-	Present	Liver enzymes	Normalization of liver enzymes, no relapses
(Bolia et al., 2016) [13]	Case series	3	14-year-old boy	Pancreatitis	Colitis	Yes	CRP = 148 mg/L	Prednisolone, AZA, UDCA	3.70 g/L (0.8—1.4)	Absent	Clinical/Radiological	Frequent relapses
			11-year-old girl	Pancreatitis	hepatitis/colitis/lymphadenopathy	Yes	-	Prednisolone, AZA, tacrolimus, MTX, infliximab	6.16 g/L	Present	Clinical/Radiological	Resolution on MTX and Infliximab
			7-year-old boy	Pancreatitis	AIHA/hepatitis	Yes	-	Prednisolone, AZA, UDCA	Normal	Present	Clinical/ LFTs/ Radiology	Normalization of LFTs, radiographic regression
(Keidar et al., 2020) [14]	Case report	1	15-year-old girl	Chronic sclerosing sialadenitis (CSS) or Küttner tumor ( left neck mass)	-	NO	-	Surgical	-	Present	-	-
(Namireddy et al., 2021) [15]	Case report	1	9-year-old girl	Fever, cough, epistaxis, nasal swelling, nasal mass	-	Yes	-	-	High	Present	-	-
(Corujeira et al., 2015) [16]	Case report	1	22-month-old female	Failure to thrive and recurrent respiratory tract infections	Multiple mediastinal lymphadenopathies,posterior mediastinal mass	No	Raised	Glucocorticoids	805 mg/dL	Present	Symptomatic, reduction in the size of the mass, and decrease of serum IgG4 levels	Symptomatic, reduction in the size of the mass, and decrease of serum IgG4 levels
(Nastri et al., 2018) [17]		1	7-year-old boy	Constitutional & skin lesions (necrotizing vasculitis, recurrent uveitis	Left kidney tumour (IgG4-RD)	Yes	-	Prednisone and azathioprine + Nephrectomy	-	Present	-	-
(Nambirajan et al., 2019) [18]	Case report	1	16-year-old male	Focal seizures large mass in the left frontoparietal region	-	-	-	Surgical	Normal	Present	-	-
(Demir et al., 2021) [19]	Case report	1	16-year-old adolescent girl	Episcleritis, palpable purpura, salivary gland enlargement, and bloody diarrhea > a focal mass in the pancreatic tail (IgG4-related AIP), GN	Renal necrotizing granulomatous vasculitis (AAV)	-	-	-	5.34 g/L (0–1.25)	Present	-	-
(Chakrabarti et al., 2019) [20]	Case report	1	9-year-old boy	FUO, PET CT scan revealed a large lobulated mass in the rectovesical pouch with increased fluorodeoxyglucose (FDG) uptake		Yes	C-reactive protein (270, 301 and 276 mg/L)	Surgical	469 mg/dL (less than 135)	Present		Resolution of fever
(Özdel et al., 2020) [21]	Case report	1	A 14-year-old girl	Swelling in the upper arm, biopsy proved IgG4-RD		No	CRP 124 mg/L, ESR 130 mm/hour	Presnisolone, MMF then Rituximab	606 mg/dL (< 135)	Present	Clincially and by APR	Resolution of mass and normalization of APR
(Akkelle et al., 2020) [22]	Case report	1	7-year-old girl	Pancreatitis and concurrent sclerosing cholangitis	IBD and lacrimal gland involvement	No		Steroids	143 mg/dL (1–108.7)	Present		
(Szczawinska-Poplonyk et al., 2016) [23]	Case report	1	7-year old atopic boy	Pneumonia, positive Epstein-Barr virus (EBV)-DNA	Posterior pulmonary consolidated mass lesion	Yes		Surgical	Normal	Present		
(Ferreira da Silva et al., 2017) [24]	Case report	1	16-year-old Hispanic male	Bilateral Submandibular swelling (IgG4-RD)		No	Normal	Prednisolone 40 mg	1050 mg/dL (< 89)	Present	Clinically	Resolution of mass after 1 year follow up
(Raab et al., 2018) [25]	Case report	1	3-year-old boy	Orbital cellulitis		No	CRP 30 mg/L	_	_	Present	_	_
(Gabrovska et al., 2021) [26]	Case report	1	17-year-old girl	Tracheal stenosis		No		Presnisolone	8.25 g/L (0.23–1.11)	Present	Clinically	
(Timeus et al., 2021) [27]	Case report	1	6-year-old boy	Left parotid swelling		No	High leucocytes	Short course dexamethaxone	Normal	Present	Clinically	Remission
(Hoshiyama et al., 2022) [28]	Case report	1	8-year-old girl	Painless upper eyelid swelling		No	-	Surgical removal	188 mg/dL (< 135)	Present	Clinically	Remission
(Tong et al., 2021) [29]	Case report	1	15-month-old boy	A homogenous mass in the left medial and inferior orbit	Immunodeficiency due to a homozygous variant in the IRAK-4 gene	No	ESR = 66 mm/hour and C-RP = 19 mg/L	Prednisone(1 mg/kg/day), AZA and trimethoprim/sulfamethoxazole prophylaxis then MMF	2.05 g/L (0–0.42)	Present	Clinically	Remission
(Kaya Akca et al., 2021) [30]	Case Series	8	14-year-old girl	Unilateral orbital swelling	Headache, proptosis	-	ESR = 7 mm/hour, CRP = 1.6 mg/L	-	1.08 g/L (0.11–1.57)	Present	-	-
(Kaya Akca et al., 2021) [30]	Case Series	8	13.6-year-old boy	Unilateral orbital swelling	Lacrimal gland swelling	-	ESR = 2 mm/hour, CRP = 1.3 mg/L	-	0.41 g/L (0.11–1.57)	Present	-	-
(Kaya Akca et al., 2021) [30]	Case Series	8	16-year-old girl	Unilateral orbital swelling	Headache	-	ESR = 27 mm/hour, CRP = 4.3 mg/L	-	1.95 g/L (0.11–1.57)	-	-	-
(Kaya Akca et al., 2021) [30]	Case Series	8	10-year-old boy	Unilateral orbital swelling	Proptosis	-	ESR = 6 mm/hour, CRP = 1.6 mg/L	-	1430 mg/dL (16–1150)	Present	-	-
(Kaya Akca et al., 2021) [30]	Case Series	8	13.3-year-old girl	Unilateral orbital swelling	Eyelid tenderness, small pulmonary nodule	Fever	ESR = 14 mm/hour, CRP = 4 mg/L	-	-	Present	-	-
(Kaya Akca et al., 2021) [30]	Case Series	8	9.3-year-old girl	Unilateral orbital swelling	Proptosis, 5^th^ cranial nerve affection	-	ESR = 50 mm/hour, CRP = 9.6 mg/L	-	0.35 g/L (0.11–1.57)	Present	-	-
(Kaya Akca et al., 2021) [30]	Case Series	8	4.2-year-old boy	Abdominal pain, mesenteric lymph node (biopsied)	-	-	ESR = 17 mm/hour, CRP = 6.4 mg/L	-	0.35 g/L (0.11–1.57)	Present	-	-
(Kaya Akca et al., 2021) [30]	Case Series	8	15.4-year-old boy	Fever, Abdominal pain	Salivary gland swelling, Ulcerative colitis, lymphadenopathy	Fever	ESR = 7 mm/hour, CRP = 1.6 mg/L	-	7.63 g/L (0.11–1.57)	Present	-	-
(Miglani et al., 2010) [31]	Case Report	1	13-year-old boy	Autoimmune pancreatitis	-	-	-	Prednisolone 20 mg /day tapered and stopped in 4 months	603 mg/dL (< 135)	Present	-	-
(Ibrahim et al., 2011) [32]	Case Report	1	3-year-old girl	Cholangitis				Prednisolone 2 mg/kg/day and AZA 1.5 mg/kg/day	258 mg/dL (< 49.1)	Present	-	Relapsed after tapering, required low dose maintenance prednisolone and AZA
(Mannion & Cron, 2011) [33]	Case Report	1	13-year-old girl	Autoimmune pancreatitis, fibrosing mediastinitis	renal and hepatic affection	-	-	Prednisolone and MMF	226 mg/dL (11–157)	Present	-	No relapse after tapering and stoppage of Prednisolone and MMF
(Zakeri & Kashi, 2011) [34]	Case Report	1	17-year-old boy	Riedel’s thyroiditis	-	-	-	Prednisolone 40 mg/ day	-	Present	-	Prednisolone tapered and stopped in 3 months
(Melo et al., 2012) [35]	Case Report	1	11-year-old boy	Sialadenitis	-	-	-	prednisolone	-	Present	-	-
(Griepentrog et al., 2013) [36]	Case Series	2	10-year-old girl	Orbital disease	-	-	-	Lateral orbitotomy	-	-	-	No further treatment needed
			14-year-old girl	Orbital disease	-	-	-	Prednisolone, MMF	-	-	-	Relapse after tapering prednisolone,, MMF was successful
(Kalapesi et al., 2013) [37]	Case Report	1	5-year-old girl	Orbital disease	-	-	-	Prednisolone, MMF	-	-		Prednisolone tapered and MMF continued successfully
(Naghibi et al., 2013) [38]	Case Report	1	16-year-old girl	Colitis	Autoimmune pancreatitis	-	-	Adalimumab	210 mg/dL (< 140)	Present	-	Refractory to Prednisolone, rituximab but responded to adalimumab
(Pifferi et al., 2013) [39]	Case Report	1	15-year-old boy	Pulmonary disease	-	-	-	Prednisolone 0.6 mg/kg/ day	1090 mg/dL (49–66)	Present	-	4 weeks
(Sane et al., 2013) [40]	Case Report	1	12-year-old girl	Orbital disease	Nephrotic syndrome	-	-	Methylprednisolone & rituximab	Normal	Present	-	Initial response but relapsed
(Caso et al., 2014) [41]	Case Report	1	17-year-old boy	Lymphadenitis	Scleritis	-	-	Prednisolone 10 mg/day & rituximab	4.43 g/L	Present	-	Refractory to MMF, but responded to Rituximab
(Hasosah et al., 2014) [42]	Case Report	1	7-year-old girl	Mesenteritis	pancreatitis	-	-	Prednisolone, AZA, colchicine	149 mg/dL (8–140)	Present	-	Relapsed on AZA, needed maintenance prednisolone
(Jariwala et al., 2014) [43]	Case Report	1	7-year-old boy	Orbital disease	-	-	-	Prednisolone, AZA	109.3 mg/dL (0.4–98)	Present	-	Good response
(Mittal et al., 2014) [44]	Case Report	1	14-year-old boy	Orbital disease	-	-	-	Prednisolone 0.6 mg/kg/day	4.39 g/L (0.049–1.985)	Present	-	Initial improvement
(Notz et al., 2014) [45]	Case Report	1	13-year-old girl	Dacroadenitis	-	-	-	Prednisolone 40 mg/day for 3 months	Normal	Present	-	-
(Prabhu et al., 2014) [46]	Case Series	2	15-year-old girl	Orbital disease	Sinonasal disease	-	-	Rituximab, insufficient response to prednisolone	206 mg/dL (6–112)	Present	-	-
			15-year-old girl	Orbital disease	-	-	-	Prednisolone	579 mg/dL (6–112)	Present	-	-
(Batu et al., 2015) [47]	Case Series	2	14-year-old girl	Orbital disease	-	-	-	Prednisolone and MTX as maintenance	7.5 g/L (0–12.5)	Present	-	-
			9-year-old girl	Orbital disease	-	-	-	Methylprednisolone & cyclophosphamide	3.7 g/L (0–12.5)	Present	-	-
(Gillispie et al., 2015) [48]	Case Report	1	7-year-old girl	Orbital disease	Renal disease	-	-	Prednisolone and rituximab	Normal	Present	-	Responded to rituximab
(Nada et al., 2015) [49]	Case Report	1	10-year-old boy	Hepatic mass	Coagulopathy	-	-	Prednisolone 2 mg/kg/day	420 mg/dL (6–28)	Present	-	Coagulopathy improved after prednisolone
(Rosen et al., 2015) [50]	Case Report	1	17-year-old boy	Cholangitis	-	-	-	Prednisolone 30 mg/ day	-	-	-	Weaned in 3 months

*Symbols:* No *ESR* Erythrocyte sedimentation rate, *CRP*: C-reactive proteins, *MTX*: Methotrexate, *APRs* Acute phase reactants, *AZA* Azathioprine, *UDCA* Ursodeoxycholic acid, *LFTs* liver function tests, *IBD* Inflammatory bowel disease, *IgG4-RD* Immunoglobulin G 4-Related Disease, *DNA* Deoxyribonucleic acid, *MMF* Mycophenolate mofetil, *IRAK-4* interleukin-1 receptor-associated kinase 4, *AIHA* Autoimmune hemolytic anemia, *AAV ANCA* Associated vasculitis, *FUO* Fever of unknown origin, *GN* Glomerulonephritis

As most of cases, a course of prednisolone and azathioprine was used as a starting regimen in our case and upon relapse rituximab was started. Along with medical treatment, pericardiotomy is a possible relieving procedure that proved to be effective in IgG4-RD with pericardial involvement.

Unfortunately, our case developed a fatal relapse unlike most of the reported cases which succeeded long remission, bearing in mind that a considerable number of the reported cases have no documented follow up.

In our case, the second biopsy, taken from the mediastinum and pleura, showed no IgG-4 positive plasma cells, probably resembling an end to the activity process by a permanent damaging fibrosis. We presume that this fibrosis eroded the bronchial blood vessels and caused such fatal hemoptysis. This presumption is supported by two facts; first of which is the normal ESR and CRP of the patient at that time, and the second is the poor response to rituximab in its second cycle.

In a literature review of 25 pediatric cases of IgG4-RD, the median age of the cases was 13 years, with 64% were girls. The predominant manifestations were IgG4-related orbital disease (44%) and autoimmune pancreatitis type 1/IgG4-related pancreatitis (12%) with pulmonary manifestations occurring in only 2 cases. Our case conforms to the average age and main gender of such cases but with a rare presentation; intrathoracic one. 24 extra cases were added to our table from this review [6].

In a recent single center experience, the researchers reviewed a total of eight patients, the details of those cases are included in the table, with a median age of 13.4 years. The manifestations were IgG4-related ophthalmic disease (six patients), IgG4-related lymphadenopathy (one patient), and IgG4-related sialadenitis and lymphadenopathy, pancreatitis, ulcerative colitis, and pulmonary manifestations (one patient). Relapse occurred in only two patients. This highlights the rarity of pulmonary involvement in pediatric age group and highlights the fact that relapse does not occur in the majority of cases [5].

As many of the searched cases (Table 1), our case was associated with systemic constitutional symptoms along with elevated inflammatory markers like ESR and CRP, so both, the constitutional manifestations and the inflammatory markers may provide non-invasive ways to monitor the disease activity and enlighten the management decisions.

In spite of discussing only orbital involvement, a review studying clinicopathological characters of orbital IgG4-RD showed absence of pathognomonic findings associated with adult version of the disease, like storiform fibrosis and obliterative phlebitis [7]. Serum IgG4 was elevated in 2 out of 4 cases. The four studied cases had treatment-responsive clinical course. This was contrary to our case which had pathognomonic histopathological features, elevated serum IgG4 and a frequently relapsing clinical course. Interestingly, the four reviewed cases had positive ANA with high titers in comparison to negative ANA in our case.

As regarding adult IgG4-RD cases with pericardial involvement, in one recent review of IgG4-RD with pericardial involvement, 32 published cases were included [8]. The mean age was 64 years and 65.7% of patients were males. IgG4-related pericarditis was mostly associated with pleural involvement, as in our case. In most cases, a pericardial biopsy was done to support the diagnosis of IgG4-RD and serum-IgG4 levels were ≥ 135 mg/dL in 86% of cases. Those findings are the same as in our case too. Most patients were initially treated with glucocorticoids, pericardiectomy or a combination of both. Only one patient was treated with rituximab as monotherapy.

To conclude: we report a case of 13-year-old female presented with a pediatric intrathoracic IgG4-RD, which is a rare form of IgG4-RD. Our case suffered from frequent relapses and ended in fatal hemoptysis, the exact cause of which has not been established. Being a disease that may affect pediatric age group, and may involve the lung, pleura and pericardium, not only the rheumatologist should be aware of this rare disease, but also the general pediatrician, the cardiologist and the pulmonologist. To our knowledge, this is the first case report to describe a case of pediatric IgG4-RD who developed fatal hemoptysis as a result of pulmonary affection.

## Data Availability

The datasets used and/or analysed during the current study are available from the corresponding author on reasonable request, anonymously.

## Abbreviations

ANA: Anti-nuclear Antibodies
APRs: Acute phase reactants
AZA: Azathioprine
CBC: Complete Blood Count
CRP: C-reactive proteins
CT: Computed tomography
DNA: Deoxyribonucleic acid
EPAP: Estimated Pulmonary Artery Pressure
ESR: Erythrocyte sedimentation rate
IBD: Inflammatory bowel disease
IgG4: Immunoglobulin G-4
IgG4-RD: Immunoglobulin G 4-Related Disease
IGRA: Interferon Gamma Releasing Assay
IRAK-4: Interleukin-1 receptor-associated kinase 4
LFTs: Liver function tests
MGIT: Mycobacteria Growth Indicator Tube
MMF: Mycophenolate mofetil
MTX: Methotrexate
RCDcriteria: Revised comprehensive diagnostic criteria
TB: Tuberculosis
UDCA: Ursodeoxycholic acid
ZN: Ziehl–Neelsen

## References

[1] Perugino C, Stone J. IgG4-related disease: an update on pathophysiology and implications for clinical care. Nat Rev Rheumatol. 2020;16(12):702-14. Available from: https://pubmed.ncbi.nlm.nih.gov/32939060/ cited 2021 Oct 9. 10.1038/s41584-020-0500-7 32939060

[2] Umehara H, Okazaki K, Kawa S, Takahashi H, Goto H, Matsui S, et al. The 2020 revised comprehensive diagnostic (RCD) criteria for IgG4-RD. Mod Rheumatol. 2021;31(3):529–33. Available from: https://pubmed.ncbi.nlm.nih.gov/33274670/ cited 2021 Oct 9. 10.1080/14397595.2020.1859710 33274670

[3] Yamamoto M, Takahashi H, Shinomura Y. Mechanisms and assessment of IgG4-related disease: lessons for the rheumatologist. Nat Rev Rheumatol. 2014;10(3):148–59. Available from: https://pubmed.ncbi.nlm.nih.gov/24296677/ cited 2021 Oct 9. 10.1038/nrrheum.2013.183 24296677

[4] Lees J, Church N, Langdale-Brown B, Bellamy C, Gibson P, Watson S. IgG4-related disease: a novel, important but easily missed condition. J R Coll Physicians Edinb. 2013;43(2):126–33. Available from: http://www.rcpe.ac.uk/journal/issue/journal_43_2/watson.pdfS. 10.4997/JRCPE.2013.208 23734354

[5] Kaya Akca Ü, Atalay E, KasapCüceoğlu M, Şener S, Balık Z, Başaran Ö, et al. IgG4-related disease in pediatric patients: a single-center experience. Rheumatol Int. 2021;2021(1):1–9. Available from: https://link.springer.com/article/10.1007/s00296-021-04885-5 cited 2021 Oct 9. 10.1007/s00296-021-04885-5 33978820

[6] Karim F, Loeffen J, Bramer W, Westenberg L, Verdijk R, van Hagen M, et al. IgG4-related disease: a systematic review of this unrecognized disease in pediatrics. Pediatr Rheumatol Online J. 2016;14(1):18. 10.1186/s12969-016-0079-3. PMC4807566 27012661

[7] Bu F, Koo SC. Clinicopathologic Characterization of IgG4-Rich Pediatric Head and Neck Lesions. Arch Pathol Lab Med. 2022;146(5):611–8. Available from: https://meridian.allenpress.com/aplm/article/146/5/611/469929/Clinicopathologic-Characterization-of-IgG4-Rich cited 2022 Jul 17. 10.5858/arpa.2021-0041-OA 34473213

[8] Doumen M, Vankelecom B, Westhovens R, Michiels S. Pericarditis as a manifestation of IgG4-related disease. Rheumatol Int. 2022;42(7):1287–95. 10.1007/s00296-021-04946-9 34255183

[9] Tille L, Schnabel A, Laass MW, Hahn G, Taut H, Leszczynska A, et al. Orbital inflammation and colitis in pediatric IgG4-related disease: A case report and review of the literature. Eur J Rheumatol. 2020;7(1):21–7. Available from: https://pubmed.ncbi.nlm.nih.gov/31804176/ cited 2021 Jun 5. 10.5152/eurjrheum.2019.19165 PMC7004272 31804176

[10] Dylewska K, Kobusinska K, Kurylak A. Tumour of the orbit and pterygopalatine fossa: Delayed recognition of possible IgG4-related disease. Wspolczesna Onkol. 2020;24(2):136–9. Available from: 10.5114/wo.2020.97638 cited 2021 Jun 5. 10.5114/wo.2020.97638 PMC7403764 32774140

[11] Smerla R, Rontogianni D, Fragoulis G. Ocular manifestations of IgG4-related disease in children. More common than anticipated? Review of the literature and case report. Clin Rheumatol [Internet]. 2018 [cited 2021 Jun 5];37(6). Available from: https://pubmed.ncbi.nlm.nih.gov/29204759/. 10.1007/s10067-017-3934-9 29204759

[12] Aydemir Y, Akcoren Z, Demir H, SaltikTemizel IN, Ozen H, Yuce A. Clinical and histopathological features of immunoglobulin G4-associated autoimmune hepatitis in children. J Gastroenterol Hepatol [Internet]. 2019;34(4):742–6. Available from: https://pubmed.ncbi.nlm.nih.gov/30378176/ cited 2021 Jun 5. 10.1111/jgh.14525 30378176

[13] Bolia R, Chong SY, Coleman L, MacGregor D, Hardikar W, Oliver MR. Autoimmune pancreatitis and IgG4 related disease in three children. ACG Case Rep J. 2016;3(4):e115. 10.14309/crj.2016.88. PMC5018227 27622194

[14] Keidar E, Shermetaro J, Kwartowitz G. Pediatric Parotid Chronic Sclerosing Sialadenitis in an African-American Female: A Rare Case and Review of the Literature. Cureus [Internet]. 2020 Jun 26 [cited 2021 Jun 5];12(6). Available from: https://pubmed.ncbi.nlm.nih.gov/32754389/. 10.7759/cureus.8846 PMC7386075 32754389

[15] Namireddy MK, Consul N, Sher AC. FDG-Avid Pulmonary Nodules and Tracheobronchial Mural Inflammation in IgG4-Related Disease. Clin Nucl Med. 2021;46(2):e125–6. 10.1097/RLU.0000000000003358 33156051

[16] Corujeira S, Ferraz C, Nunes T, Fonseca E, Vaz LG. Severe IgG4-Related Disease in a Young Child: A Diagnosis Challenge. Case Rep Pediatr. 2015;2015:1–4. 10.1155/2015/140753 PMC4325228 25705537

[17] Nastri MMF, Novak GV, Sallum AEM, Campos LMA, Teixeira RAP, Silva CA. Immunoglobulin G4-related disease with recurrent uveitis and kidney tumor mimicking childhood polyarteritis nodosa. Acta Reumatol Port. 2018;2018(3):226–9. Available from: https://pubmed.ncbi.nlm.nih.gov/30414371/ cited 2021 Oct 9. 30414371

[18] Nambirajan A, Sharma MC, Garg K, Sriram S, Boorgula MT, Suri V. Large dural-based mass with bony hyperostosis in a 16-year-old male: IgG4-related disease mimicking lymphoplasmacyte-rich meningioma. Child’s Nerv Syst. 2019;35(8):1423–7. 10.1007/s00381-019-04187-z 31073682

[19] Demir AM, Aydin F, Acar B, Kurt T, Poyraz A, Kiremitci S, et al. IgG4-related disease and ANCA positive vasculitis in childhood: a case-based review. Clin Rheumatol. 2021;40(9):3817–25. 10.1007/s10067-021-05635-5 33590421

[20] Chakrabarti S, Pal P, Dutta S, Nada R. IgG4-related Disease at Rectovesical Pouch Mimicking Inflammatory Myofibroblastic Tumor. Indian Pediatrics. 2019;56:1059–61. Available from: https://www.indianpediatrics.net/dec2019/dec-1059-1061.htm. cited 2021 Oct 9. 31884443

[21] Özdel S, Ekim M, Kaygusuz G, Çelikel E, Vatansever G, Taçyıldız N. A new location for pediatric immunoglobulin G4 related disease: The biceps muscle. Turk J Pediatr. 2020;62(3):495–7. 10.24953/turkjped.2020.03.020 32558427

[22] Akkelle BŞ, Tutar E, Ergelen R, Çelikel ÇA, Ertem D. IgG4 related disease in a seven year old girl with multiple organ involvement: A rare presentation. Turk Pediatr Ars. 2020;55(2):191–4. Available from: http://www.ncbi.nlm.nih.gov/pubmed/32684765 cited 2021 Oct 6. 10.14744/TurkPediatriArs.2019.83435 PMC7344129 32684765

[23] Szczawinska-Poplonyk A, Wojsyk-Banaszak I, Jonczyk-Potoczna K, Breborowicz A. Pulmonary manifestation of immunoglobulin G4-related disease in a 7-year-old immunodeficient boy with Epstein-Barr virus infection: a case report. Ital J Pediatr. 2016;42(1):58. 10.1186/s13052-016-0269-0. PMC4898369 27277432

[24] Ferreira da Silva RC, Lieberman SM, Hoffman HT, Policeni B, Bashir A, Smith RJH, et al. IgG4-related disease in an adolescent with radiologic-pathologic correlation. Radiol Case Reports. 2017;12(1):196–9. 10.1016/j.radcr.2016.10.008 PMC5310240 28228909

[25] Raab EL, Moayedpardazi HS, Naids SM, Friedman AH, Meltzer MA. Lacrimal gland abscess in a child as a rare manifestation of IgG4-related disease. J AAPOS. 2018;22(1):73-75.e1. 10.1016/j.jaapos.2017.08.013 29288833

[26] Gabrovska N, Velizarova S, Spasova A, Kostadinov D, Yanev N, Shivachev H, et al. A Case of Tracheal Stenosis as an Isolated Form of Immunoproliferative Hyper-IgG4 Disease in a 17-Year-Old Girl. Child (Basel, Switzerland). 2021;8(7). Available from: http://www.ncbi.nlm.nih.gov/pubmed/34356568 cited 2021 Oct 7. 10.3390/children8070589 PMC8307327 34356568

[27] Timeus F, Calvo MM, Caci AM, Gallone GO, Vittone F. IgG4-related chronic sclerosing sialadenitis in a child with recurrent parotitis: a case report. BMC Pediatr. 2021;21(1):1–7. Available from: https://bmcpediatr.biomedcentral.com/articles/10.1186/s12887-021-03004-4 cited 2022 Jul 17. 10.1186/s12887-021-03004-4 PMC8691005 34930210

[28] Hoshiyama S, Maruyama Y, Iwaya M, Uehara T, Nakazawa Y. Immunoglobulin G4-related orbital disease in an 8-year-old girl. Pediatr Int. 2022;64(1):e15014. Available from: https://onlinelibrary.wiley.com/doi/full/10.1111/ped.15014 cited 2022 Jul 17. 10.1111/ped.15014 35278255

[29] Tong JY, Leahy KE, Wong M, Krivanek M, Tumuluri K. IgG4-related disease of the orbit in an infant. J Am Assoc Pediatr Ophthalmol Strabismus {JAAPOS}. 2021;25(4):255–7. Available from: http://www.jaapos.org/article/S1091853121001488/fulltext cited 2022 Jul 17. 10.1016/j.jaapos.2021.04.005 34166820

[30] Kaya Akca Ü, Atalay E, KasapCüceoğlu M, Şener S, Balık Z, Başaran Ö, et al. IgG4-related disease in pediatric patients: a single-center experience. Rheumatol Int. 2021;42(7):1177–85. Available from: https://link.springer.com/article/10.1007/s00296-021-04885-5 cited 2022 Jul 17. 10.1007/s00296-021-04885-5 33978820

[31] Miglani RK, Murthy D, Bhat R, Kumar AKV. Immunoglobulin G4-associated cholangitis mimicking cholangiocarcinoma in a young boy. J Postgrad Med. 2010;56(2):140–2. 10.4103/0022-3859.65294 20622394

[32] Ibrahim SH, Zhang L, Freese DK. A 3-year-old with immunoglobulin g4-associated cholangitis. J Pediatr Gastroenterol Nutr. 2011;53(1):109–11. 10.1097/MPG.0b013e31820e82a1 21694546

[33] Mannion M, Cron RQ. Successful treatment of pediatric IgG4 related systemic disease with mycophenolate mofetil: case report and a review of the pediatric autoimmune pancreatitis literature. Pediatr Rheumatol Online J [Internet]. 2011 Jan 4 [cited 2022 Aug 29];9(1). Available from: https://pubmed.ncbi.nlm.nih.gov/21205323/. 10.1186/1546-0096-9-1 PMC3022838 21205323

[34] Zakeri H, Kashi Z. Variable Clinical Presentations of Riedel’s Thyroiditis: Report of Two Cases. Case Rep Med [Internet]. 2011 [cited 2022 Aug 29];2011. Available from: https://pubmed.ncbi.nlm.nih.gov/21837243/. 10.1155/2011/709264 PMC3152964 21837243

[35] Melo JC, Kitsko D, Reyes-Múgica M. Pediatric chronic sclerosing sialadenitis: Küttner Tumor. Pediatr Dev Pathol. 2012;15(2):165–9. 10.2350/11-04-1023-OA.1 21985393

[36] Griepentrog GJ, Vickers RW, Karesh JW, Azari AA, Albert DM, Bukat CN. A clinicopathologic case study of two patients with pediatric orbital IgG4-related disease. Orbit. 2013;32(6):389–91. 10.3109/01676830.2013.822899 23957841

[37] Kalapesi FB, Garrott HM, Moldovan C, Williams M, Ramanan A, Herbert HM. IgG4 orbital inflammation in a 5-year-old child presenting as an orbital mass. Orbit. 2013;32(2):137–40. 10.3109/01676830.2013.764449 23480695

[38] Naghibi M, Ahmed A, al Badri AM, Bateman AC, Shepherd HA, Gordon JN. The successful treatment of IgG4-positive colitis with adalimumab in a patient with IgG4-related sclerosing disease--a new subtype of aggressive colitis? J Crohns Colitis. 2013;7(3):e81–4. 10.1016/j.crohns.2012.05.003. 22647639

[39] Pifferi M, Di Cicco M, Bush A, Caramella D, Chilosi M, Boner AL. Uncommon pulmonary presentation of IgG 4-related disease in a 15-year-old boy. Chest. 2013;144(2):669–71. 10.1378/chest.12-3088 23918112

[40] Sane M, Chelnis J, Kozielski R, Fasiuddin A. Immunoglobulin G4-related sclerosing disease with orbital inflammation in a 12-year-old girl. J AAPOS. 2013;17(5):548–50. 10.1016/j.jaapos.2013.05.017 24160980

[41] Caso F, Fiocco U, Costa L, Sfriso P, Punzi L, Doria A. Successful use of rituximab in a young patient with immunoglobulin G4-related disease and refractory scleritis. Jt Bone Spine. 2014;81(2):190–2. 10.1016/j.jbspin.2013.07.011 23932926

[42] Hasosah MY, Satti MB, Yousef YA, Alzahrani DM, Almutairi SA, Alsahafi AF, et al. IgG4-related sclerosing mesenteritis in a 7-year-old Saudi Girl. Saudi J Gastroenterol. 2014;20(6):385–8. 10.4103/1319-3767.145333 PMC4271016 25434322

[43] Jariwala MP, Agarwal M, Mulay K, Sawhney S. IgG4-Related Orbital Inflammation Presenting as Unilateral Pseudotumor. Indian J Pediatr. 2014;81(10):1108–10. 10.1007/s12098-014-1471-y 24854367

[44] Mittal R, Ganguly A, Rath S, Das B, Mishra A. IgG4-related orbital inflammation presenting as bilateral proptosis in a child. Eye. 2014;28(10):1264–6. 10.1038/eye.2014.154 PMC4194328 24993327

[45] Notz G, Intili A, Bilyk JR. IgG4-related dacryoadenitis in a 13-year-old girl. Ophthal Plast Reconstr Surg. 2014;30(6):e161–3. 10.1097/IOP.0000000000000016 24814270

[46] Prabhu SM, Yadav V, Irodi A, Mani S, Varghese AM. IgG4-related disease with sinonasal involvement: A case series. Indian J Radiol Imaging. 2014;24(2):117–20. 10.4103/0971-3026.134384 PMC4094959 25024517

[47] Batu ED, Arici ZS, Orhan D, Kiratli H, Ozen S. Immunoglobulin G4-related orbital disease: A report of two paediatric cases. Clin Exp Rheumatol. 2015;33(3):409–10. Available from: https://www.clinexprheumatol.org/abstract.asp?a=8735 cited 2022 Aug 29. 25897575

[48] Gillispie MC, Thomas RD, Hennon TR. Successful treatment of IgG-4 related sclerosing disease with rituximab: A novel case report. Clin Exp Rheumatol. 2015;33(4):549–50. Available from: https://www.clinexprheumatol.org/abstract.asp?a=8689 cited 2022 Aug 29. 25897489

[49] Nada R, Gupta A, Kang M, Rawat A, Sood A, Ahluwalia J, et al. Hepatic mass and coagulopathy in a ten-year-old boy with fever. Arthritis Rheumatol. 2015;67(7):1977. 10.1002/art.39131 25809271

[50] Rosen D, Thung S, Sheflin-Findling S, Lai J, Rosen A, Arnon R, et al. IgG4-sclerosing cholangitis in a pediatric patient. Semin Liver Dis. 2015;35(1):89–94. 10.1055/s-0034-1398475 25632939

